# First person – Daniel Bronder

**DOI:** 10.1242/dmm.049319

**Published:** 2021-11-30

## Abstract

First Person is a series of interviews with the first authors of a selection of papers published in Disease Models & Mechanisms, helping early-career researchers promote themselves alongside their papers. Daniel Bronder is first author on ‘ *TP53* loss initiates chromosomal instability in fallopian tube epithelial cells’, published in DMM. Daniel conducted the research described in this article while a doctoral student in Thomas Ried and Stephen Taylor's labs at the National Cancer Institute, Bethesda, MD, USA and the University of Manchester, Manchester, UK. He is now a postdoctoral fellow in the lab of Samuel Bakhoum at Memorial Sloan Kettering Cancer Center, New York, NY, USA, investigating the causes and consequences of chromosomal instability in cancer.


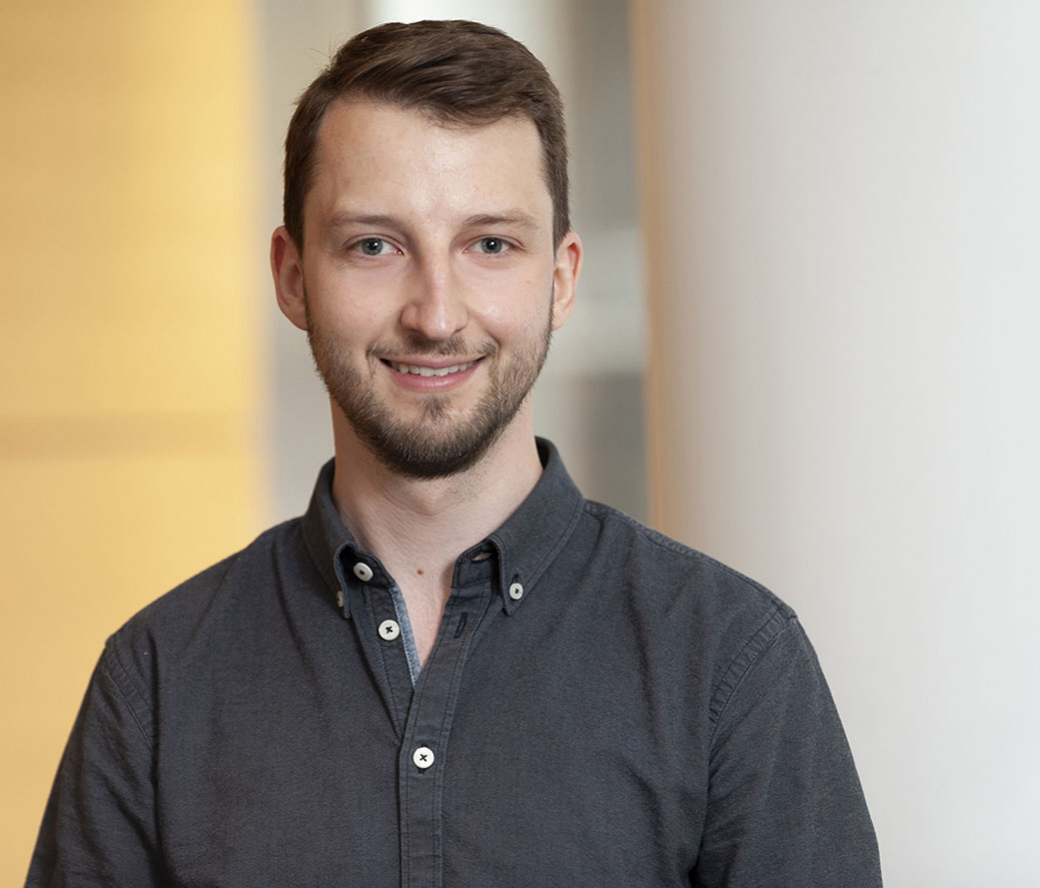


**Daniel Bronder**

**How would you explain the main findings of your paper to non-scientific family and friends?**

Cell division is happening constantly in the human body. Just before a cell divides, the DNA, which is organized into chromosomes, must be duplicated first and then divided equally between two daughter cells. In cancer cells, this otherwise robust process goes awry, which leads to constant errors in cell division and an imbalance of chromosome number between the daughter cells. This is what we call chromosomal instability. For my doctoral thesis work, I investigated if changes in the DNA sequence of specific genes (i.e. gene mutations) would cause errors in cell division and the consequential changes in chromosome number in daughter cells. Ultimately, we could show that changing the DNA sequence of two genes strongly associated with cancer (*TP53* and *BRCA1*) resulted in increased errors during cell division and chromosome number abnormalities.

“[…] changing the DNA sequence of two genes strongly associated with cancer (*TP53* and *BRCA1*) resulted in increased errors during cell division and chromosome number abnormalities.”

**What are the potential implications of these results for your field of research?**

Our data imply that mutating a single gene, albeit a very important one, is sufficient to cause an imbalance in chromosome numbers in an otherwise near-normal cell. Interestingly, p53 is only indirectly involved in controlling cell division by regulating the expression of mitotic factors. This leads to the question if there is one specific or multiple mitotic factors downstream of p53, which become deregulated in its absence, thus causing aberrations in cell division. While attempts have been made in the past, this observation precipitates the possibility to identify mitotic targets that are deregulated in p53-deficient cancers specifically for therapeutic intervention. That way, cancer cells could be targeted selectively sparing p53-proficient, healthy cells.

**What are the main advantages and drawbacks of the model system you have used as it relates to the disease you are investigating?**

A critical step in the beginning of this project was to ensure that the pathways we were interested in studying were intact in our model system of choice. We chose FNE1 cells as these cells were immortalized using only one ectopic construct, a human telomerase transgene. We then confirmed pathway integrity by various means, which allowed us to subsequently dissect the role of *TP53*, *BRCA1* and *MYC* with high confidence as intrinsic deregulation of these factors had pre-emptively been ruled out. These points are clear advantages of our system; however, its *in vitro* nature limits other aspects that might have been of interest to our readers. For instance, it remains unclear what role the immune system plays in limiting the proliferation of cells that have experienced an aberrant cell division. To answer questions in that realm, a more sophisticated system is required that combines the culture of epithelial cells with immune cells such as T-, B- or natural killer cells.

**What has surprised you the most while conducting your research?**

I was most surprised by our observation that loss of p53 alone precipitated chromosome segregation defects. The landmark studies investigating p53 in that context had previously failed to make said observation. Seeing that change in our own hands was really striking. Above that, I was really impressed by the consistency we saw. Especially as we were doing revision experiments, we had a sense of what to expect, but the three key experiments we performed really fell into place and strengthened our story overall.

**Figure:**
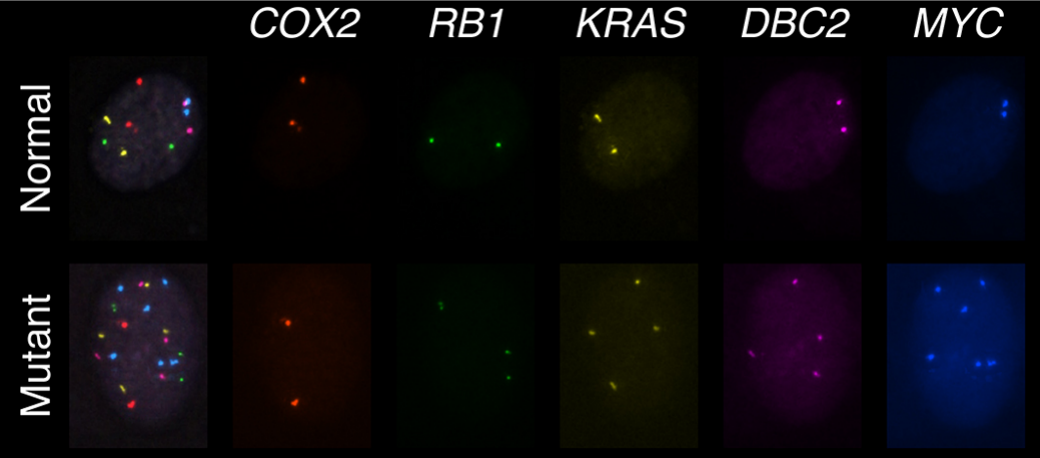
A normal and mutant fallopian tube cell are shown to illustrate gene copy number changes at five loci, as determined by multiplex interphase fluorescence *in situ* hybridization.

**Describe what you think is the most significant challenge impacting your research at this time and how will this be addressed over the next 10 years?**

Chromosomal instability and aneuploidy in cancer is a really exciting field to be part of. Over many years, the drivers were either cell biologists investigating mitotic fidelity in a bottom-up approach or pathologists as well as oncologists looking at it in a top-down manner. However, merging these two approaches has only recently begun. The continued decrease in next-generation sequencing cost and advancement in more user-friendly analytical tools will have brought that on in part. In my opinion, we need to build on that momentum and keep bringing expertise from both areas together and train the next generation of experts in both to improve our understanding of chromosomal instability and aneuploidy in cancer. Ultimately, that way, we will be able to capitalize on our expanded understanding of failed cell division for the benefit of cancer patients.

“Chromosomal instability and aneuploidy in cancer is a really exciting field to be part of.”

**What changes do you think could improve the professional lives of early-career scientists?**

In my mind, the most common response to this question is likely going to be ‘more funding’. So, I will highlight something else that early-career scientists would greatly benefit from. Especially in the USA, the health care, social and retirement benefits are diverse and opaque. Despite that, many institutes provide these kinds of benefits for graduate students and postdocs, but I have found these groups are unaware or uninformed about how to best utilize these tools. Therefore, I would wish for a better effort of institutions in providing us with some level of coaching on how to access these resources within or outside of the institute and maximize what we can get out of them.

**What's next for you?**

A few weeks after we submitted our manuscript to Disease Models & Mechanisms, I moved from Bethesda, where I had lived for 2.5 years, to New York. Here, I started a postdoctoral fellowship at Memorial Sloan Kettering Cancer Center, where I am continuing my work on chromosomal instability in cancer. Even after 6 months, I am still filled with excitement every day to have become part of such a vibrant cancer research community. In my new project, I am focusing on *in vivo* models of chromosomal instability and its role in therapy response and resistance.
